# A Roadmap for Future Parkinson's Pharmacogenomics in Asia

**DOI:** 10.3389/fnagi.2022.896371

**Published:** 2022-05-17

**Authors:** Muhammad Akbar, Gita Vita Soraya, Zulvikar Syambani Ulhaq, Andi Kurnia Bintang

**Affiliations:** ^1^Department of Neurology, Faculty of Medicine, Hasanuddin University, Makassar, Indonesia; ^2^Department of Biochemistry, Faculty of Medicine, Hasanuddin University, Makassar, Indonesia; ^3^Research Center for Pre-Clinical and Clinical Medicine, National Research and Innovation Agency Republic of Indonesia, Cibinong, Indonesia

**Keywords:** Parkinson's disease, pharmacogenomics, Asia, genetics, polymorphism

## Introduction

Parkinson's disease (PD) remains one of the most debilitating neurodegenerative diseases, with increasing prevalence worldwide. Due to the demographic transition ongoing in many countries, life expectancy is increasing, whilst chronic and degenerative conditions such as Parkinson's also rise with aging (Dorsey et al., 2018). The disease is typically seen in persons 60 years of age or older who are already more likely to experience additional burden due to limited physical, cognitive, and emotional capacity in their day-to-day lives. However, it is notable that earlier onset of the disease have been identified in a small subset of patients (Post et al., 2020). When left untreated, PD can exert a negative long-term impact on the quality of life of both the patient and the caregiver (Asimakopoulos et al., 2008).

Although the exact pathogenesis of the disease remains elusive, it has been suggested that it develops as a results of dopaminergic neuron degeneration in the substansia nigra (Schulz-Schaeffer, 2010). Pharmacotherapy still relies on traditional agents such as levodopa, despite the emergence of novel agents and elective surgical interventions such as deep brain stimulation that has been shown to provide long-term improvement of motor function (Limousin and Foltynie, 2019). Levodopa remains the most clinically-effective and cost-effective Parkinon's disease treatment, which can provide an adequate success rate in the majority of cases upon administration that considers the most optimal bioavailability and efficacy. However, a subset of patients may experience the occurrence of motor levodopa-induced complications, including levodopa-induced dyskinesia or motor fluctuations, rendering the levodopa treatment ineffective (Soraya et al., 2022).

Although Asia had the largest number of PD incidence in 2019, a lower prevalence of PD has been documented in Japan and Singapore, despite having a relatively high proportion of aged individuals. Moreover, it is notable that sex-related differences have been reported in PD prevalence, clinical phenotypes, and prognosis. Hence, these findings pinpoint that genetic and environmental risk factors may play an important role to the differences observed in the region.

## Pharmacogenetics and PD

Over the past few decades, we have seen rapid development of genetic explorations in PD, mainly due to advances in genotyping technology such as next generation sequencing, in addition to the rising number of genome wide association studies. Initially, genetic exploration in PD aimed to identify causative genetics or the genetic architecture of the condition, with first discoveries focused on the autosomal dominant and recessive mutations in PD (SCNA, LRRK2, VPS35 and PINK1, DJ-1, Parkin genes) and environment-gene interactions in the pathobiology of PD (Lill, 2016). The work has since extended in to pharmacogenetic profiling in PD, especially regarding the genetic basis of levodopa induced complications to determine whether certain gene polymorphisms can increase an individual's chance of experiencing ineffective levodopa treatment (Espay et al., 2018; Kalinderi et al., 2019; Falla et al., 2021; Soraya et al., 2022). This is supported by improved bioinformatics tools such as the PharmCAT (Pharmacogenomics Clinical Annotation Tool) (Klein and Ritchie, 2018), or pharmacogenetic and pharmacogenomic databases such as the Pharmacogenomics Knowledge Base (PharmGKB) (Hewett et al., 2002), and the CPIC database (Relling and Klein, 2011) that provide peer-reviewed information on gene/drug pairs.

Hence, numerous gene polymorphisms have been associated with the occurrence of these motor complications, a majority of which are involved in the dopamine metabolic and signaling pathways, including polymorphisms of the dopamine transporter/solute carrier-6 (DAT/SLC6) genes responsible for trans-membrane dopamine transport (Purcaro et al., 2019), and those that encode dopamine receptor families D1 (DRD1, DRD5) and D2 (DRD2, DRD3, DRD4) (Rieck et al., 2012; Kaplan et al., 2014; Dos Santos et al., 2019). Interestingly, a large subset of these gene polymorphisms are not only highly prevalent in Asians, but also demonstrate stronger associations in the Asian population. An example is the COMT rs4680 and rs4633 which in our previous meta-analysis was found to be markers of dyskinesia in Asian ethnicities (Soraya et al., 2022). Together, these imply that genetic aspects are crucial in order to elucidate regional characteristics.

It is clear that in this new era of PD management, we are equipped with technological support and research advancements on PD pharmacogenetics. Which then brings us to the question of what can be done next to accelerate the clinical implementations? Whilst many predict that implementation is still a long road ahead, particularly in resource poor settings, there is definitely a high likelihood of translation, seeing the success of previous pharmacogenetic screening practices. Therefore, it is vitally important to understand an inter-ethnic difference as the fields enters the era of genetics-based targeted therapies.

## Discussion

In this opportunity, we would like to propose a roadmap for future implementation of Parkinsons's pharmacogenomics particularly in Asia. Firstly, more clinical trials need to be performed to confirm the associations using pharmacogenetic endpoints as the outcome, such as the occurrence of LID or MF. There are several issues with current evidence, mainly due to the limited sample size and the clinical heterogeneity that subjects present with. The potential variance across populations require careful consideration, and we propose that the trials for Asian populations need to be performed and also consider the differences between Asian ethnicities. In fact, the establishment of initiatives such as the South East Asian Pharmacogenomics Research Network (SEAPharm) by member countries Korea, Indonesia, Malaysia, Taiwan, and Thailand can be valuable in assisting the strengthening pharmacogenomics research and the advancement of pharmacogenetics testing within the region (Chumnumwat et al., 2019).

Aside from improving the evidence on clinical robustness, more effort needs to be done in assessing cost-effectivity. In particular, how does the cost of testing fare against the cost of treating side effects and against the cost of additional patient visits or hospital stays. Several studies have assessed the cost-effectivity of pharmacogenetic testing for implementation of warfarin pharmacogenetic testing (Ma and Lu, 2011) and for carbamazepine hypersensitivity (Chen et al., 2016). These illustrate that more effort need to be made to realize strategies to improve the cost-effectivity of implementation. Along with assessing clinical validity and cost-effectivity, we also need to initiate awareness of the pharmacogenetics and precision medicine, not only amongst physicians but also for patients. Moreover, discussions should also continue regarding the ethical and social issues associated with implementation of pharmacogenetic testing in the disease population.

The acquirement of robust evidence then needs to be supported by guidelines from regulatory bodies regarding the pharmacogenetic profile of levodopa. This reflects previously successful pharmacogenomics screening such as HLA-B^*^15:02 for carbamazepine hypersensitivity, in which the pharmacogenetic side effect was clearly listed by the FDA. Currently, several consortiums have been set up to speed translation and supervise recommendations, such as the Clinical Pharmacogenetics Implementation Consortium, (CPIC), which are capable of releasing guidelines regarding the clinical implantation (Relling and Klein, 2011).

In conclusion, a united effort is needed to speed up the clinical implementation of pharmacogenomics in PD, especially in low resource settings such as Asia. This involves synchronous effort to improve clinical validity, increase accessibility to affordable genotyping platforms, establish cost-effectivity, and the design and implementation of appropriate regulations. Moreover, epidemiological as well as genetic studies in the Asian region would better advance understanding of PD characteristics in order to develop better approaches to overcome PD.

## Author Contributions

All authors listed have made a substantial, direct, and intellectual contribution to the work and approved it for publication.

## Conflict of Interest

The authors declare that the research was conducted in the absence of any commercial or financial relationships that could be construed as a potential conflict of interest.

## Publisher's Note

All claims expressed in this article are solely those of the authors and do not necessarily represent those of their affiliated organizations, or those of the publisher, the editors and the reviewers. Any product that may be evaluated in this article, or claim that may be made by its manufacturer, is not guaranteed or endorsed by the publisher.
